# Molecular study of specific virulence genes of *Pseudomonas aeruginosa* isolated from sub clinical bovine mastitis cases

**DOI:** 10.1038/s41598-026-69514-2

**Published:** 2026-09-10

**Authors:** Eman S. Ibrahim, Sohad M. Dorgham, Rasha Hamdy Eid, Eman F. Abdel-latif, Doaa D. Khalaf

**Affiliations:** 1 Department of Microbiology and Immunology, National Research Centre, Dokki, Giza, 12622 Egypt; 2 Udder Health and Neonatal Disease, Animal Reproduction Research Institute, Giza, Egypt; 3 Department of Food Hygiene & Control, Faculty of Veterinary Medicine, Cairo University, Giza, 12211 Egypt

**Keywords:** *Pseudomonas aeruginosa*, Mastitis, Virulence genes, Diseases, Microbiology, Molecular biology

## Abstract

*Pseudomonas aeruginosa*-caused bovine mastitis has received increased attention recently because it results in large financial losses for dairy farms and the dairy sector overall. *P. aeruginosa* mastitis is a complex illness that requires host, pathogen, and environmental interactions. The objective of this study was to examine the distribution and presence of virulence factors in 13 *Pseudomonas aeruginosa* isolates that were isolated from 70 milk samples from subclinical cases of bovine mastitis, with an incidence of 18.57%. The Vitek 2 system (bioMerieux) verified each isolate. *Pseudomonas aeruginosa* produces virulence factors, which give it significant roles in the cause of the disease These virulence factors are encoded by virulence genes found on the *Pseudomonas* chromosome. The *ecfx* gene was identified in 13 (100%) of the 13 *P. aeruginosa* isolates, according to molecular identification of the bacteria and detection of specific virulence genes. Additionally, all of the current genes*) lasR*,* rhlI*,* pqsR*,* lecA*,* lasA*,* proC*,* toxA*,* exoS*,* and pelA*) were found in 6 out of 13 isolates with an incidence of 46.15%.

## Introduction

Mastitis is one of the most prevalent and fatal diseases in dairy production, causing significant financial losses for the dairy sector each year. A variety of etiological factors, particularly bacterial infections, can cause mastitis^1^. *Pseudomonas aeruginosa (P. aeruginosa)* is an important opportunistic pathogen associated with subclinical bovine mastitis, where it utilizes a variety of pathogenic pathways that involve several virulence genes^2,3^.

Moreover, due to its innate ability to survive for extended periods of time in both dry and moist situations, *P. aeruginosa* is commonly identified in the surrounds of cattle, because it needs minimal resources to develop and replicate, it is widely distributed in the environment of dairy cows^4^. In dairy farms, it has been found in water supplies from all sources, including sprinkler pens, ponds, wells, troughs, and wash rooms. It has the ability to induce a sudden outbreak of clinical mastitis in dairy farms in a matter of days, regardless of weather and/or management conditions^5^.

The pathogenesis of *P. aeruginosa* is influenced by a variety of virulence factors. The development of alternative therapeutic techniques to monitor and prevent these *Pseudomonas* infections depends on a knowledge of the regulatory mechanisms controlling the expression of virulence genes due to the variety of pathways for virulence^6^. Different morphological and biochemical characterizations were used to identify *P. aeruginosa*, and then *ecfX* gene-specific PCR-based identification was used as a dependable genetic marker of *P. aeruginosa*^7^. Additionally, *ecfX* gene encodes an ECF (extra cytoplasmic function) sigma factor of *P. aeruginosa*, and which may contribute to virulence^8^.

*Pseudomonas* infections are frequently challenging to treat because quorum sensing (QS) controls the formation of biofilms and bacterial activity^9^. *P. aeruginosa* has two primary QS systems, *las* and *rhl*. Each system consists of two components, the auto inducer synthases (*lasI* and *rhlI*, respectively) and their cognate transcriptional regulators (*lasR* and *rhlR*, respectively)^10^. In fact, antibiotic susceptibility is decreased by 100–1000 times once a biofilm forms^11^. The coordination of several hierarchical signaling networks is what is causing this rise in antibiotic resistance: On the one hand, (QS) (Las/Rhl/Pqs) controls a cascade control of extracellular matrix elements including Pel/Psl polysaccharides and eDNA-rhamnolipid complexes, as well as virulence factors like elastase LasB and alginate synthase AlgD^12^. This cooperation creates a two-pronged defense system that includes both a physical barrier and an improved resistance gene transfer mechanism^13^.

Numerous extracellular and cell-associated virulence factors have been identified in *P. aeruginosa*. Protein synthesis is inhibited by exotoxin A, a significant virulence component of *P. aeruginosa* that is encoded by the *toxA* gene. In fact, Exotoxin A (toxA) and exoenzyme S (exoS), which are encoded by the exoS gene, are two key lethal weapons that are still linked to subclinical mastitis infection in cows^14^. This study’s goal was to identify the phenotypic and genotypic characteristics of isolated *P. aeruginosa* strains from mastitic cows through detection of specific virulence genes (*Ecfx*,* Ias R*,* rhll*,* pqsR*,* Lec A*,* IasA*,* ProC*,* toxA*,* exoS*,* PelA*).

## Materials and methods

### Samples collection

A total of 70 raw milk samples were collected from dairy cows suffering from subclinical mastitis, diagnosed according to the California Mastitis Test (CMT) and Somatic Cell Count > 200,000 cells/mL according to José et al. (2018), with no history of prior antimicrobial therapy during the current lactation cycle. These animals belonged to various smallholder farms distributed across the Giza and Monofia governorates in Egypt. Before any sampling took place, the objectives of the study were thoroughly explained to the herd owners, who subsequently provided their voluntary informed consent. Approximately 10 mL of milk was aseptically collected from each animal during the morning milking session (8:00–10:00 a.m.) and transferred into sterile Falcon tubes. To ensure sample integrity and minimize contamination, the teat ends were carefully disinfected prior to sampling, and stringent hygiene guidelines were maintained throughout the process, with a complete assurance that the animals were not subjected to any invasive or experimental procedures.

### Isolation and identification of *P. aeruginosa*

The milk samples were plated onto *Pseudomonas* agar plates at 37 °C for 24 h. The suspected colonies were purified and cultured onto nutrient agar slopes and incubated at 37 °C for 24 h. The purified colonies were subjected for further identification either morphologically and biochemically according to Prescott et al.^15^. The purified isolates of *P. aeruginosa* were subsequently confirmed using a Vitek 2 system (bioMe′rieux) which is rapid and reliable identification method^16^.

### Molecular detection of certain virulence genes

#### DNA extraction

Genomic DNA extraction was performed on all 13 confirmed *P. aeruginosa* isolates following the manufacturer’s instructions for the DNeasy Blood & Tissue Kit (Qiagen, Germany). All bacterial isolates were screened for the presence of *ecfx gene* specific for *P. aeroginosa* and also for virulence *genes* (*Ias R*,* rhll*,* pqsR*,* Lec A*,* IasA*,* ProC*,* toxA*,* exoS*,* PelA*).

PCR reactions were performed using SimpliAmp™ Thermal Cycler (Cat. No. A24811, Applied Biosysytems, USA) in a final volume of 25 µl reaction containing 12.5 µl of 2x MyTaq™ Red Mix Master Mix (Cat. BIO-25043, Meridian Bioscience, UK), 0.5 µl (10 µM) of each primer and 1 µl of target DNA. The PCR products were separated by electrophoresis on 1.5% agarose gel then photographed and analyzed by InGenius3 gel documentation system (Syngene, UK). Cycling conditions for gene detection in this study are listed in Tables 1 and 2. In every PCR run, a positive control (*P. aeruginosa* ATCC 27853) and a negative control (nuclease-free water) were included to ensure reaction validity.

**Table 1:** PCR primers utilized in the study.

Gene	Sequence (5′-3′)	Amplicon size (bp)	References
*Ecfx*	ATGGATGAGCGCTTCCGTG TCATCCTTCGCCTCCCTG	528 bp	^17^
*Ias R*	AAGTGGAAAATTGGAGTGGAG GTAGTTGCCGACGATGAAG	130 bp	^18^
*Rhll*	GTAGCGGGTTTGCGGATG CGGCATCAGGTCTTCATCG	101 bp	^19^
*pqsR*	CTGATCTGCCGGTAATTGG ATCGACGAGGAACTGAAGA	142 bp	^19^
*Lec A*	CACCATTGTGTTTCCTGGCGTTCA AGAAGGCAACGTCGACTCGTTGAT	101 bp	^20^
*IasA*	TTCTGTGATCGATTCGGCTCGGTT ACCCGGGAAGACAACTATCAGCTT	106 bp	^20^
*ProC*	CAGGCCGGGCAGTTGCTGTC GGTCAGGCGCGAGGCTGTCT	180 bp	^21^
*toxA*	GACAACGCCCTCAGCATCAACAGC CGCTGGCCCATTCGCTCCAGC	397 bp	^22^
*exoS*	AGGCATTGCCCATGACCTTG ATACTCTGCTGACCTCGCTC	372 bp	^22^
*PelA*	AAGAACGGATGGCTGAAGG TTCCTCACCTCGGTCTCG	149 bp	^22^

**Table 2:** Cycling conditions for the genes detection in this study.

Gene	Init. Denat.	Denat.	Anneal.	Extension	Final Ext.	Cycles
*Ecfx*	94 °C 2 min	94 °C 30 s	58 °C 30 s	72 °C 45 s	72 °C 7 min	35
*Ias R*	94 °C 2 min	94 °C 30 s	60 °C 30 s	72 °C 45 s	72 °C 7 min	35
*Rhll*	94 °C 2 min	94 °C 30 s	58 °C 30 s	72 °C 45 s	72 °C 7 min	35
*pqsR*	94 °C 2 min	94^°^C 30 s	58 °C 30 s	72 °C 45 s	72 °C 7 min	35
*Lec A*	94 °C 2 min	94 °C 30 s	56 °C 30 s	72 °C 45 s	72 °C 7 min	35
*IasA*	94 °C 2 min	94 °C 30 s	56 °C 30 s	72 °C 45 s	72 °C 7 min	35
*ProC*	94 °C 2 min	94 °C 30 s	60 °C 30 s	72 °C 45 s	72 °C 7 min	35
*toxA*	94 °C 5 min	94 °C 30 s	65 °C 30 s	72 °C 45 s	72 °C 7 min	35
*exoS*	94 °C 5 min	94 °C 30 s	60 °C 30 s	72 °C 45 s	72 °C 7 min	35
*PelA*	94 °C 5 min	94 °C 30 s	60 °C 30 s	72 °C 45 s	72 °C 7 min	35

## Results

### Isolation and identification of *P. aeruginosa*

Thirteen isolates (18.57%) recovered from the 70 milk samples produced characteristic growth features of *Pseudomonas* species on the cetrimide agar medium. The isolates were obtained as one specific colony. The isolates were confirmed by Vitek 2system (bioMe’rieux).

### Molecular identification of *P. aeruginosa* and detection of certain virulence genes

In the current study, the *ecfx* gene was recognized in 13 (100%) out of 13 *P. aeruginosa* isolates. Moreover, all the present genes were detected in the same set of 6 out of 13 isolates with an incidence 46.15% (*ias R*,* rhll*,* pqsR*,* lec A*,* iasA*,* proC*,* toxA*,* exoS*,* pelA* ).

**Fig. 1:**
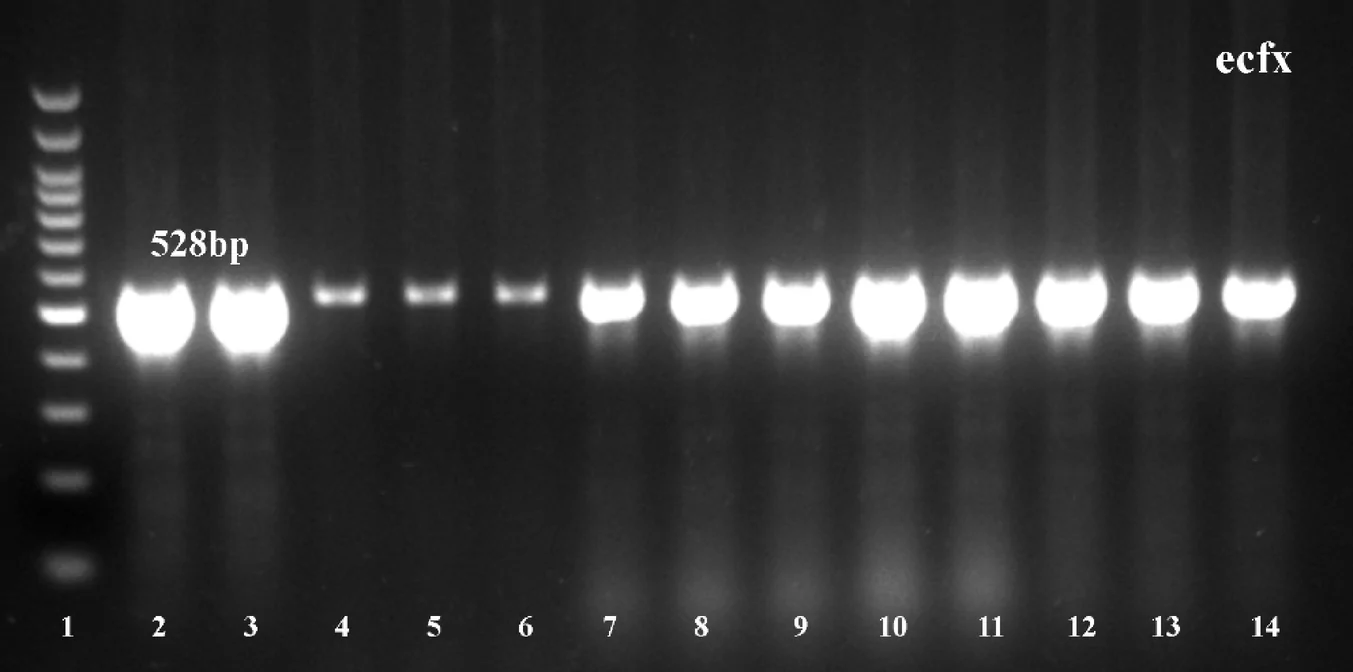
Agarose gel electrophoresis of PCR product amplified from *P.aeroginosa ecfx* gene (528 bp). Lane 1, 100 bp DNA Ladder; Lanes 2–14, positive samples.

All the bacterial isolates (13) were confirmed as *P. aeruginosa* using PCR targeting *ecfx* gene as shown in Fig. 1.

**Fig. 2:**
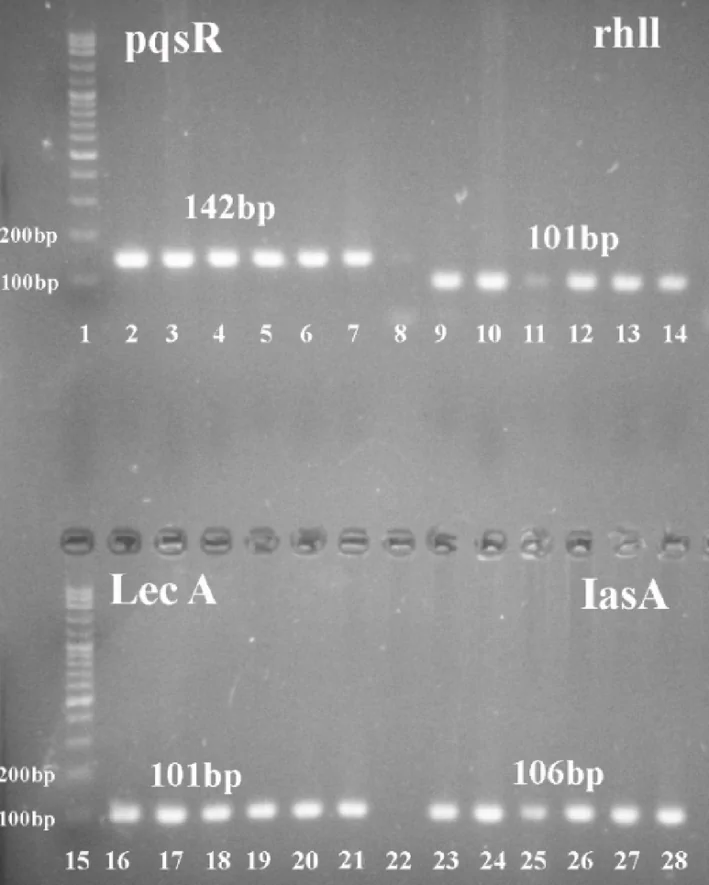
Agarose gel electrophoresis of PCR product amplified from representative *P. aeroginosa pqsR* gene (Lanes 2–7), *rhII* gene (lanes 9–14), *lecA* gene ( Lanes 16–21), *iasA* gene (Lanes 23–28), Lanes 1 &14, 100 bp DNA Ladder.

**Fig. 3:**
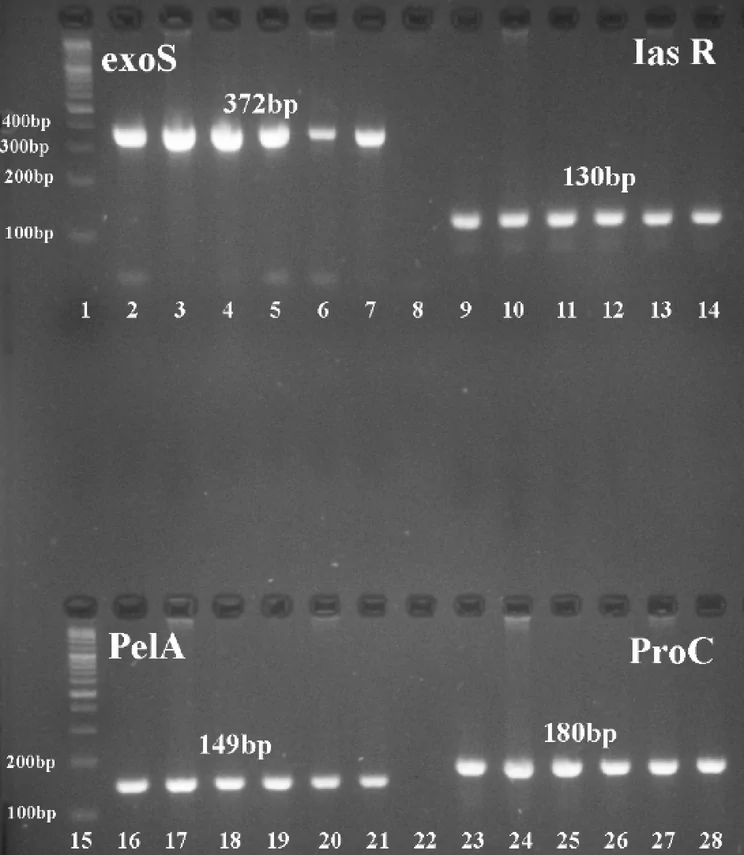
Agarose gel electrophoresis of PCR product amplified from representative *P. aeroginosa exoS* gene (Lanes 2–7), *ias R* gene (lanes 9–14), *pelA* gene ( Lanes 16–21), *ProC* gene (Lanes 23–28), Lanes 1 &14, 100 bp DNA Ladder.

**Fig. 4:**
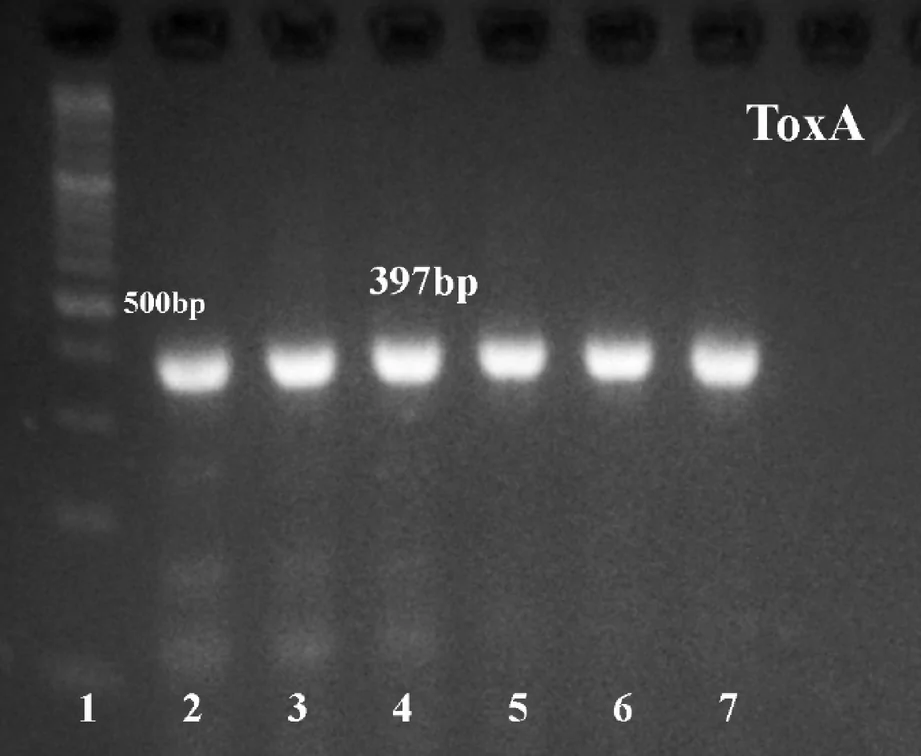
Agarose gel electrophoresis of PCR product amplified from *P. aeroginosa ToxA* gene (397 bp). Lane 1, 100 bp DNA Ladder; Lanes 2–7, representative positive samples.

All *P. aeroginosa* isolates (13) were positive in PCR targeting all virulence genes as represented in Figs. 2, 3 and 4.

Furthermore, the complete panel of tested virulence genes (*lasR*,* rhlI*,* pqsR*,* lecA*,* lasA*,* proC*,* toxA*,* exoS*,* and pelA*) was concurrently detected in the same set of 6 isolates (46.15%). The prevalence and biological functions of all target virulence genes among the isolated strains are summarized in Table 3.

**Table:** 

Target gene	Biological function	No. of positive isolates	Percentage (%)
*ecfX*	Extra-cytoplasmic function sigma factor (Species Identification)	13	100.0
*lasR*	Quorum sensing regulator (*las* system)	6	46.15
*rhlI*	Autoinducer synthase (*rhl* system)	6	46.15
*pqsR*	*Pseudomonas* quinolone signal regulator	6	46.15
*lecA*	Cytotoxic lectin PA-IL	6	46.15
*lasA*	Elastase gene	6	46.15
*proC*	Pyrroline-5-carboxylate reductase (Housekeeping/Metabolic)	6	46.15
*toxA*	Exotoxin A production	6	46.15
*exoS*	Exoenzyme S (Type III secretion effector)	6	46.15
*pelA*	Pel polysaccharide synthesis (Biofilm matrix)	6	46.15

## Discussion

One of the most frequent causes of subclinical mastitis in dairy cattle is *P. aeruginosa*, which is principally the reason for the significant financial losses faced by dairy farms^23^. The results of our investigation, which identified 13 *P. aeruginosa* isolates from 70 milk samples with an incidence of 18.57%, were consistent with those of^24^ who obtained 10 isolates of *P. aeruginosa* from 57 cows with chronic mastitis with an incidence of 17.54%.

*P. aeruginosa’s* pathogenesis is influenced by a wide range of various virulence factors and regulatory genes. Due of its significant sensitivity and specificity, *ecfX* gene has been suggested as a target for *P. aeruginosa* identification^25^. In the current study, *ecfX* gene was detected in all *P. aeruginosa* isolates.

According to our study, all of these specific virulence genes (*lasR*,* rhlI*,* pqsR*,* lecA*,* lasA*,* proC*,* toxA*,* exoS*,* and pelA*) were concurrently detected in 6 out of 13 *P. aeruginosa* isolates with an incidence of 46.15%. In comparison, previous study reported variable detection rates for key virulence determinants, with *exoS*,* toxA*, and *lasR* frequencies ranging from 16.7% to 88.9%^24^. Similarly, another study demonstrated that *P. aeruginosa* isolates possessed pathogenic genes including exoS (36.8%) and toxA (63.2%)^26^.

While *P. aeruginosa* strains showed Virulence factors screening revealed the presence of *exoS*,* toxA* and *lasR* genes with the detection rates ranging from 16.7 to 88.9%^24^. It was shown that these bacteria carried pathogenic genes such exoS (36.8%) and toxA (63.2%)^26^.

In Egypt, *P. aeruginosa* possesses a variety of virulence factors that may contribute to its pathogenicity. In contrast to our findings where ecfX was present in 100% of the isolates, a previous studystated that *toxA* genes was detected in all 6 tested strains of *P. aeruginosa*, while *lasI*,* Rhl1*and ExoS genes detected only in 3 isolates, whereas the ecfX gene was not detected in any of their strains^27^. Furthermore, *toxA*, *exoS*, and *lasB* genes were reported in 100%, 83.3%, and 66.6% of *P. aeruginosa* isolates, respectively isolated from bovine mastitis milk^23^.

Because the *exoS* gene may be more important than previously believed in tissue necrosis and inflammatory reactions, where Exoenzyme S acts as a Type III secretion effector that disrupts host cellular architecture, the vast majority of *P. aeruginosa* strains containing *exoS* must be regarded as potentially dangerous to dairy cows^28^. Similarly, Exotoxin A (encoded by *toxA*) inhibits host cellular protein synthesis, promoting tissue destruction. There are several discrepancies between our findings and those of a recent study^29^, which reported that *exoS* gene was only found in one strain, the lasA gene had the lowest detection rate of 28.57%, and The detection rates of the remaining virulence genes (*toxA*,* lasR* and *rhlL*) ranged from 65% to 81%.

In actuality, Quorum Sensing (QS), a cell density-dependent mechanism, controls the majority of *P. aeruginosa* virulence factors and orchestrates the expression of extracellular enzymes^30^. A previous investigation demonstrated that all isolates had various combinations of QS system genes, auto inducers (PAI-1 and PAI-2) biosynthesis and upregulation of virulence genes^2^; these findings were in harmony with our study.

According to another report^31^, there is a strong correlation between the presence of specific virulence genes and the formation of biofilms. The molecular detection for virulence factors associated with *P. aeruginosa* genes revealed that 86.4%, 80. %, and 74% of the biofilm-producing isolates harbored the *pel A*,* toxA*,* and exoS* genes, respectively. The *pelA* gene plays an essential role in synthesizing the primary exopolysaccharide matrix required for biofilm structural integrity, which impairs antimicrobial penetration. Similar outcomes were previously reported, demonstrating that strains which produced biofilms had high expression of the pel A gene (80%)^32^. Additionally, all isolates of *P. aeruginosa* that produce biofilms include the *pel A* gene^33^.

Lastly, as we found nearly every virulence gene associated with the formation of biofilms, our results were corroborated by all earlier studies.

## Conclusion

While *P. aeruginosa* is frequently regarded as an environmental organism, it acts as a significant cause of subclinical mastitis in dairy cattle. Persistence in subclinical form allows the pathogen to establish long-term biofilm-mediated colonization without exhibiting severe clinical symptoms. Usually, contaminated water used to bathe the udder is the source of this infection. Under stressful circumstances and inadequate hygiene, this bacterium colonizes in milking equipment, enters teats during milking intervals, and causes mastitis.

More studies employing different strains of *P. aeruginosa* are needed to show the potential link between biofilm formation and the expression of virulence genes. This is crucial because it is a direct cause of survival in animal environments, particularly milking farms, where mastitis will ultimately result.

## Data Availability

All data generated or analyzed during this study are included in this published article.
